# Numbers of Beauty: An Innovative Aesthetic Analysis for Orthognathic Surgery Treatment Planning

**DOI:** 10.1155/2016/6156919

**Published:** 2016-01-19

**Authors:** Tito Matteo Marianetti, Giulio Gasparini, Giulia Midulla, Cristina Grippaudo, Roberto Deli, Daniele Cervelli, Sandro Pelo, Alessandro Moro

**Affiliations:** ^1^Department of Maxillofacial Surgery, Catholic University Medical School, Via Pier Luigi Galletti 3, 00135 Rome, Italy; ^2^Department of Orthodontics, Catholic University Medical School, Rome, Italy

## Abstract

The aim of this study was to validate a new aesthetic analysis and establish the sagittal position of the maxilla on an ideal group of reference. We want to demonstrate the usefulness of these findings in the treatment planning of patients undergoing orthognathic surgery. We took a reference group of 81 Italian women participating in a national beauty contest in 2011 on which we performed Arnett's soft tissues cephalometric analysis and our new “Vertical Planning Line” analysis. We used the ideal values to elaborate the surgical treatment planning of a second group of 60 consecutive female patients affected by skeletal class III malocclusion. Finally we compared both pre- and postoperative pictures with the reference values of the ideal group. The ideal group of reference does not perfectly fit in Arnett's proposed norms. From the descriptive statistical comparison of the patients' values before and after orthognathic surgery with the reference values we observed how all parameters considered got closer to the ideal population. We consider our “Vertical Planning Line” a useful help for orthodontist and surgeon in the treatment planning of patients with skeletal malocclusions, in combination with the clinical facial examination and the classical cephalometric analysis of bone structures.

## 1. Introduction

The surgery in the treatment of facial deformities is based on skeletal movements of jaw bones. The movements must be carefully planned because even small displacements have highly significant influence on the final aesthetic result. The surgical treatment is based on cephalometric analysis done on lateral cephalometric radiographs. The cephalometric analysis differs from surgeon to surgeon because of their subjective evaluation. The aim is always to determine in detail the spatiality of the splanchnocranium and to compare the values obtained with the values defined “standard” for race, gender, and age. The comparison between the two values determine the skeletal movements that can be exploited to achieve the best possible result.

Most cephalometric analyses are based on skeletal data but the best results are obtained on analysis based on soft tissue data [1–8].

Today, this aesthetic analysis, providing exact data ranges for several characteristics of the soft tissue profile, is still the most employed analysis in the visual treatment planning of orthognathic patients [9, 10]. This is witnessed by the fact that modern software for orthognatic Visual Treatment Planning is based on Arnett's STCA.

During the repeated use of the STCA on patients with prognathism we found a flaw in Arnett's brilliant analysis: for those surgeons who perform their jaws surgery starting from Le Fort I osteotomy, how is it possible to assess the precise position that the upper jaw has to take with surgery? How can we assess the precise position that the upper jaw has to take if the reference line “true vertical line” origins from “Subnasale,” which is a soft tissue point the surgeon moves during maxillary displacement with Le Fort 1 osteotomy? [11, 12].

Arnett's STCA does not specify the exact amount of maxillary advancement required in cases of maxillary retrusion. Therefore we realized the importance of locating the reference line on a point the surgeon will not move during bimaxillary surgery [13] in order to identify the exact and ideal position of Arnett's soft tissue landmarks, especially the point Subnasale, which we consider expression of the sagittal position of the upper jaw. As a fix reference landmark, we decided to take the soft tissue Glabella, through which we draw our new “Vertical Planning Line,” perpendicular to the natural head position.

The aim of the present study is to validate a new aesthetic analysis and to establish the sagittal position of the maxilla on an ideal group of reference. We also want to demonstrate the usefulness of these findings in the treatment planning of patients undergoing orthognathic surgery for the correction of the sagittal position of the jaw.

## 2. Material and Methods

**Figure 1:**
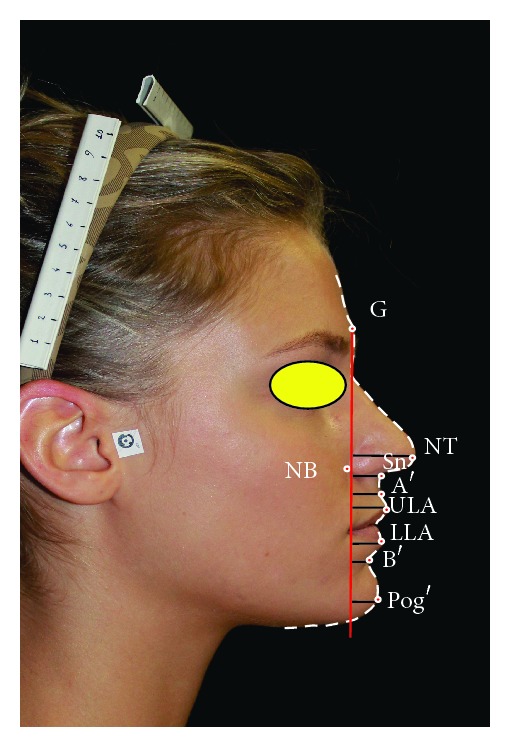
“Vertical Planning Line” analysis on a participant to “Miss Italia 2011.”

In order to validate this new aesthetic analysis, we took a reference group consisting of 81 Italian attractive women participating in a national beauty contest in 2011. Every participant has given his consent to participate in this study. The women aged between 18 and 25 years, with an average age of 21 years and 6 months, were selected among thousands of other participants. Their jaw relationship was not considered. A standardized frontal and profile photograph was taken for every participant (Figure 1).

This group of women underwent Arnett's soft tissue analysis with the Dolphin software 9.5 on lateral photographs taken with the subjects in natural head position, that is, the position obtained with the subject standing and looking at his reflection in a mirror positioned exactly at eye level.

We calculated the mean and standard deviation of each parameter of Arnett's STCA to find out if the group fits in Arnett's proposed norms.

Later on we drew our new “Vertical Planning Line” on the reference group passing through soft tissue Glabella (G′), the most prominent point on the forehead. We measured the distances in millimetres of the following landmarks from the VPL. We calculated means and standard deviations for all landmarks considered:

1. Nasal tip (NT): most prominent point on the tip of the nose.
2. Nasal base (NB): the deepest point next to the alar base.
3. Subnasale (Sn): where the labial philtrum meets the base of the nose.
4. Soft tissue A′ point (A′): the most concave point of the philtrum.
5. Upper lip anterior (ULA): the most anterior point of the upper lip mucosa.
6. Lower lip anterior (LLA): the most prominent point of the lower lip mucosa.
7. Soft tissue B′ point (B′): the most concave point on the labiomental sulcus.
8. Soft tissue Pogonion (Pog′): the most convex point of the chin profile.

Another group of 60 female patients, aged between 18 and 40, affected by skeletal class III malocclusion who came to our observation during the period from October 2011 to May 2012 was recruited for our study and analysed with our method. Exclusion criteria were congenital syndromes with craniofacial involvement, cleft lip and palate, scars in the maxillomandibular region, and asymmetries on the frontal and vertical planes.

The treatment planning for these patients was than assessed on the basis of the classical cephalometric analysis integrated with the data we got from our new “Vertical Planning Line” analysis, considering the soft tissue changes occurring after orthognathic surgery (soft/hard tissue ratio) using the following formula for each soft tissue landmark:

(1) $$\begin{array}{c} \text{BM}=\text{RV}-d\left(\;\text{VPL},\text{STL}\;\right):x. \end{array}$$

 BM stands for Bone Movement, the distance in mm the bone has to be moved.
 RV is the reference value measured on the reference group.

*d*(VPL, STL) is the distance from our “Vertical Planning Line” to the soft tissue landmark considered. The *x* value is the ratio of effective soft tissue changes that occur after a certain amount of skeletal movement. Scientific literature configured different values for this soft/hard tissue ratio [14, 15]. As other authors [14, 15], we used Epker and Fish's [16] prediction of the soft tissue changes after maxillary advancement and mandibular setback.

Considering “Subnasale” (Sn), the point from where we begin our treatment planning, Epker and Fish [16] indicate a ratio of 0.5 between the skeletal A′ point and soft tissue Sn. Hence, for example, if the point Sn is located at a distance of 7 mm from the VPL, it is 1.5 mm behind the ideal value 8.5 mm. The surgeon will perform a 3 mm advancement of the maxilla considering our equation:

(2) RV−dVPL,STL:x=BM8.5 mm−7 mm:0.5=3 mm.

All patients underwent bimaxillary orthognathic surgery with Le Fort I osteotomy for maxillary advancement and bilateral sagittal split osteotomy for mandible setback. Sixteen patients also required genioplasty.

**Figure 2:**
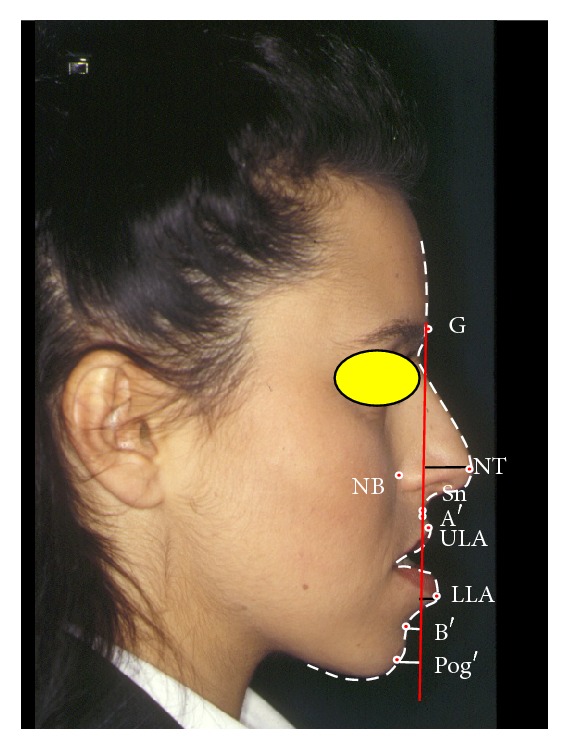
“Vertical Planning Line” analysis on a woman with skeletal class III malocclusion before orthognathic surgery.

**Figure 3:**
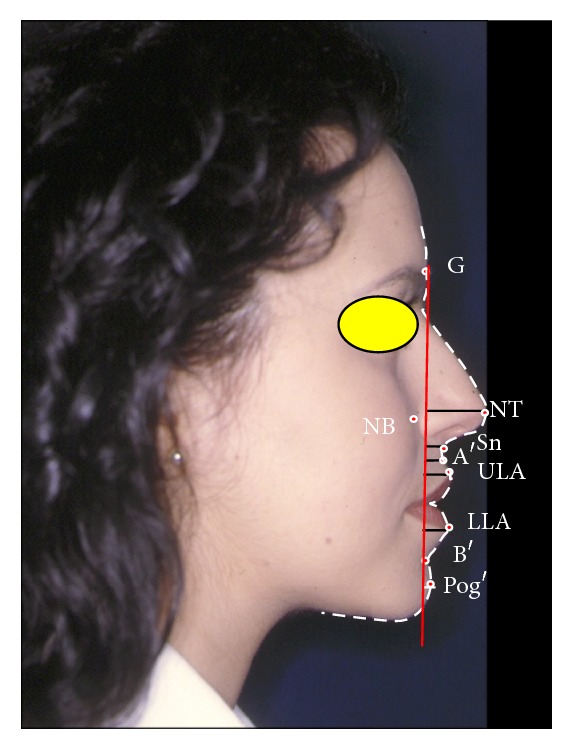
“Vertical Planning Line” analysis on the same subject of Figure 1 after orthognathic surgery.

The patients entered then a postoperative follow-up program, with photographic and clinical controls after 2 weeks, 1 month, 3 months, and 6 months (Figures 2 and 3).

At the 6-month control we repeated our new aesthetic analysis on the right profile picture.

**Figure 4:**
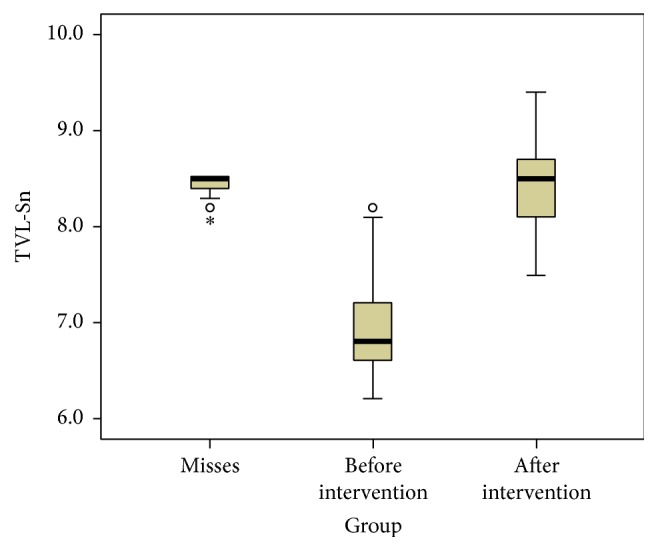
Box plot showing the distribution of the TVL-Sn values in the models population and in the subject who underwent maxillofacial surgery, before and after the surgical intervention. Values in mm.

**Figure 5:**
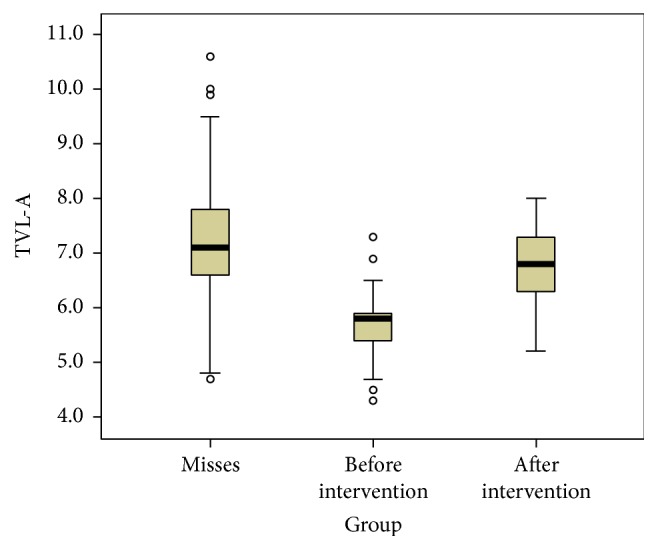
Box plot showing the distribution of the TVL-A values in the models population and in the subject who underwent maxillofacial surgery, before and after the surgical intervention. Values in mm.

**Figure 6:**
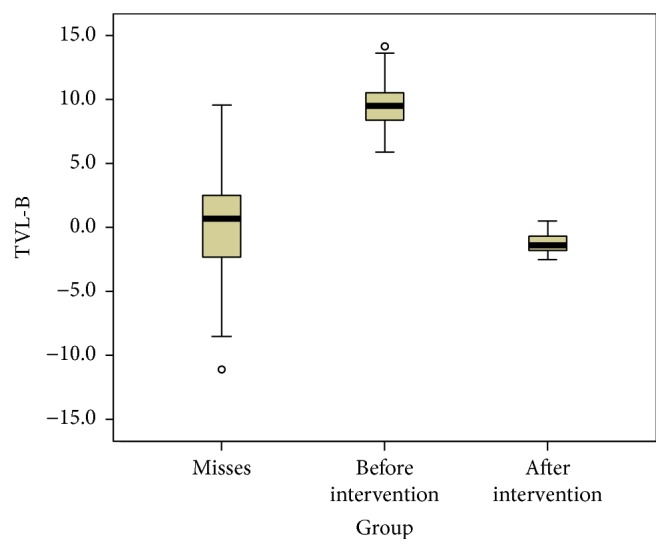
Box plot showing the distribution of the TVL-B values in the models population and in the subject who underwent maxillofacial surgery, before and after the surgical intervention. Values in mm.

Finally we compared the patients' values before and after orthognathic surgery with the reference values we got from the group of women participating in the beauty contest using box plots for all soft tissue landmarks (Figures 4, 5, and 6).

To prevent interobserver error, all processes (landmark identification and linear measurements) were performed by one author and were repeated twice during a 2-week interval.

*Statistical Analysis*. We performed a descriptive analysis of the samples included in this study reporting the means and the standard deviations of the observed quantitative variables. Box plots were used to describe the distributions of the observed values for each segment. Student's *t*-test was used to assess the presence of significant differences between Arnett's original population and the Italian girls participating in the beauty contest according to Arnett's analysis. The analysis was performed using SPSS software version 12.0 for Windows and statistical significance level was set at *p* ≤ 0.01.

## 3. Results

The comparison of the reference group of girls participating in the beauty contest with Arnett's proposed norms with the Student *t*-test showed significant statistical differences (*p* < 0.01) for the following parameters: interlabial gap, upper lip thickness, lower lip thickness, orbital rim, subpupil, soft tissue A′ point, upper lip anterior, lower lip anterior, soft tissue B′ point, soft tissue Pogonion, facial angle, forehead to maxilla, forehead to chin, orbital rim to maxilla, Subnasale to chin, maxilla to mandible, and lip to lip (Table 1).

The means and standard deviations of the landmarks' distances from the VPL measured on the reference group are illustrated in Table 2.

Regarding the data on the patients with maxillomandibular malformations observed from October 2011 to May 2012, Table 3 shows the means and standard deviations of all patients before and after orthognathic surgery.

From the descriptive statistical comparison of the patients' values before and after orthognathic surgery with the reference values we observed how all parameters considered got closer to the ideal population (Figures 3, 4, and 5).

## 4. Discussion

The study of patients with skeletal malformations often represents a challenge in the daily clinical practice.

The difficulties of the surgical treatment planning of patients affected by skeletal deformities of the jaw are due to the great number of cephalometric analysis methods and the difficulty of choosing an ideal standard population as reference.

From a recent review of the scientific literature and the mass media trends relating to the subject, we can infer there is a growing interest in aesthetic and appearance [14–18]. This tendency brought modern orthodontists to elaborate cephalometric analysis of the soft tissue profile to better study their patients. Arnett proposed his own STCA, as a result of several studies about the soft tissues profile. Nowadays this STCA is still the most employed, in the diagnosis and treatment planning of orthodontic-surgical patients [9, 10].

In order to define ideal aesthetic standards for the study of women with skeletal deformity of the jaws, we decided to study a large group of Italian attractive women, selected by the Italian population for a national beauty contest.

From the statistical analysis of our results a significant difference was found between Arnett's proposed values and our reference group. Arnett used a group of 26 adult caucasic models chosen for their good clinical characteristics to define his soft tissue cephalometric values, whereas our group consists of 81 women representative of the current Italian ideal of beauty, selected among the population by a national beauty contest. Our group should not be considered a standard reference group, but an ideal population.

Using Arnett's STCA for the routinely treatment planning of patients affected by jaw malformations, we found a flaw. This analysis does not provide the exact amount of maxillary advancement required in cases of maxillary retrusion. So how can the surgeon who moves first the upper maxilla plan his intervention in cases presenting maxillary hypoplasia, without a fix landmark of reference?

In the attempt to answer these questions we began working on a new aesthetic analysis based on the “Vertical Planning Line” passing through the point Glabella (G′), a fixed landmark that the surgeon will not move during surgery. First of all, this new reference line provides standard values on the large reference population. In addition to that, the fixed landmark G′ allows planning in advance the repositioning of any point of the soft tissue profile, always considering the different distance between the landmark and the VPL and the hard/soft tissue ratio, as explained with the formula in Materials and Methods.

Furthermore the innovative advantage of this fix landmark lays in the fact that pre- and postsurgical profile pictures can easily be compared, both between each other and with the reference values.

The comparison of pre- and postsurgical pictures of skeletal class III patients with the ideal population demonstrated how orthognathic surgery brought the patient's values closer to the ideal population, obtaining an improvement in facial balance.

Such an analysis could be used also to assess the ideal position of the nose tip, as well as the upper and lower lip anterior projection in those cases where the imperfections could be corrected by ancillary surgery such as genioplasty, rhinoplasty, or zygomatic augmentation or just with the use of fillers.

## 5. Conclusions

We emphasize that Arnett's soft tissue cephalometric analysis still remains a fundamental tool in the treatment planning of orthognathic surgery. We tried to fulfill Arnett's STCA with our new vertical line, passing through the soft tissue Glabella, a point not influenced by bimaxillary surgery, to define better the sagittal position of the upper jaw.

In fact using an ideal group of attractive Italian women, selected during a beauty contest among thousand participants we have now a value defining the ideal sagittal position of the “Subnasale” point, which shows a minimal standard deviation (±0.1 mm) in the reference population.

We consider our Vertical Planning Line a useful help for orthodontist and surgeon in the treatment planning of patients with maxillomandibular malocclusions, to integrate the clinical facial examination and the classical cephalometric analysis of bone structures.

## Figures and Tables

**Table 1:** Arnett's STCA on the reference group.

Variable	Mean Misses	SD Misses	Arnett's Mean	Arnett's SD	*p*
Facial heights					
Upper lip length (mm)	21.3	±2.5	21	±1.9	n.s.
Interlabial gap (mm)	1.2	±0.7	3.3	±1.3	<0.001
Lower lip length (mm)	44.9	±4.7	46.9	±2.3	n.s.
Lower 1/3 of face (mm)	67.3	±6.7	71.1	±3.5	n.s.
Facial height (mm)	118.3	±16.6	124.6	±4.7	n.s.
Soft tissue thickness					
Upper lip thickness (mm)	16.1	±2.9	12.6	±1.8	<0.001
Lower lip thickness (mm)	11.3	±1.9	13.6	±1.4	<0.001
Projections to TVL					
Glabella (mm)	−8.5	±0.1	−8.5	±2.4	n.s.
Orbital rim (mm)	−22.1	±3.4	−18.7	±2.0	<0.001
Cheekbone (mm)	−21.6	±4.1	−20.6	±2.4	n.s.
Subpupil (mm)	−17.6	±2.6	−14.8	±2.1	<0.001
Nasal projection (mm)	15.2	±2.4	16	±1.4	n.s.
Alar base (mm)	−12.9	±2.2	−12.9	±1.1	n.s.
Soft tissue A′ (mm)	−1.2	±1.2	1	±1.7	<0.001
Upper lip anterior (mm)	1.6	±1.6	3.7	±1.2	<0.001
Upper lip angle (°)	8.7	±7.9	12.1	±5.1	n.s.
Nasolabial angle (°)	108.9	±14	103.5	±6.8	n.s.
Lower lip anterior (mm)	−1.8	±2.7	1.9	±1.4	<0.001
Soft tissue B′ (mm)	−8.3	±3.6	−5.3	±1.5	<0.001
Soft tissue Pogonion′ (mm)	−5.9	±4.4	−2.6	±1.9	<0.001
Throat length (mm)	55.1	±8.2	58.2	±5.9	n.s.
Facial harmony					
Facial angle (°)	166.5	±4.6	169.3	±3.4	<0.001
Forehead to maxilla (mm)	7.2	±1.2	8.4	±2.7	0.002
Forehead to chin (mm)	2.6	±4.4	5.9	±2.3	0.001
Orbital rim to maxilla (mm)	20.9	±3.2	18.5	±2.3	0.001
Orbital rim to chin (mm)	16.3	±4.5	16	±2.6	n.s.
Subnasale to chin (mm)	5.9	±4.4	3.2	±1.9	0.006
Maxilla to mandible (mm)	7.1	±3.0	5.2	±1.6	0.005
Lip to lip (mm)	3.3	±1.9	1.8	±1.0	0.006
Lower lip to chin (mm)	4.1	±2.8	4.5	±2.1	n.s.
B′ to chin (mm)	2.5	±1.8	2.7	±1.1	n.s.

**Table 2:** New “Vertical Planning Line” on the reference group.

Variable	Misses mean	Misses SD
VPL-NT (mm)	23.7	±2.4
VPL-NB (mm)	−4.5	±2.2
VPL-Sn (mm)	8.5	±0.1
VPL-A′ (mm)	7.2	±1.2
VPL-ULA (mm)	10	±1.7
VPL-LLA (mm)	6.7	±2.6
VPL-B′ (mm)	0.1	±3.5
VPL-Pog′ (mm)	2.6	±4.4

**Table 3:** New “Vertical Planning Line” on the group of class III patients before and after surgery (SD in brackets).

Variable	Patient's mean before surgery	Patients' mean after surgery
VPL-NT (mm)	22.4 (±1.2)	23.2 (±0.9)
VPL-NB (mm)	−8.2 (±0.8)	−4.4 (±0.4)
VPL-Sn (mm)	5.6 (±0.8)	8.4 (±0.4)
VPL-A′ (mm)	4.9 (±0.8)	6.7 (±0.7)
VPL-ULA (mm)	6.4 (±0.8)	8.7 (±0.5)
VPL-LLA (mm)	15.9 (±1.9)	5.7 (±0.8)
VPL-B′ (mm)	9.6 (±1.7)	−1.3 (±0.7)
VPL-Pog′ (mm)	12.9 (±1.8)	1.2 (±0.9)

## Abbreviations

VPL: “Vertical Planning Line”
G′: “Glabella”
NT: Nasal tip
NB: Nasal base
Sn: Subnasale
A′: Soft tissue A′ point
ULA: Upper lip anterior
LLA: Lower lip anterior
B′: Soft tissue B′ point
Pog′: Soft tissue Pogonion
BM: Bone Movement
RV: Reference value
*d*(VPL, STL): The distance from our “Vertical Planning Line” to the soft tissue landmark considered
*x* value: The ratio of effective soft tissue changes that occur after a certain amount of skeletal movement
Sn: Subnasale
Arnett's STCA: Arnett's soft tissue cephalometric analysis.

## References

[1] Kamoen A., Dermaut L., Verbeeck R. (2001). The clinical significance of error measurement in the interpretation of treatment results. *European Journal of Orthodontics*.

[2] Adenwalla S. T., Kronman J. H., Attarzadeh F. (1988). Porion and condyle as cephalometric landmarks-an error study. *American Journal of Orthodontics and Dentofacial Orthopedics*.

[3] Iglesias-Linares A., Yáñez-Vico R.-M., Moreno-Manteca B., Moreno-Fernández A. M., Mendoza-Mendoza A., Solano-Reina E. (2011). Common standards in facial esthetics: craniofacial analysis of most attractive black and white subjects according to people magazine during previous 10 years. *Journal of Oral and Maxillofacial Surgery*.

[4] Auger T. A., Turley P. K. (1999). The female soft tissue profile as presented in fashion magazines during the 1900s: a photographic analysis. *The International Journal of Adult Orthodontics and Orthognathic Surgery*.

[5] Baker B. W., Woods M. G. (2001). The role of the divine proportion in the esthetic improvement of patients undergoing orthodontic/orthognathic surgical treatment. *The International Journal of Adult Orthodontics and Orthognathic Surgery*.

[6] Dürer A. (1528). *Vier Bücher Über Menschlicher Proportion*.

[7] Alberti L. B. (1972). *“On Painting” and “On Sculpture”*.

[8] Butow K.-W. (1984). A lateral photometric analysis for aesthetic-orthognathic treatment. *Journal of Maxillofacial Surgery*.

[9] Arnett G. W., Jelic J. S., Kim J. (1999). Soft tissue cephalometric analysis: diagnosis and treatment planning of dentofacial deformity. *American Journal of Orthodontics and Dentofacial Orthopedics*.

[10] Arnett G. W., Bergman R. T. (1993). Facial keys to orthodontic diagnosis and treatment planning. Part I. *American Journal of Orthodontics and Dentofacial Orthopedics*.

[11] Legan H. L., Burstone C. J. (1980). Soft tissue cephalometric analysis for orthognathic surgery. *Journal of Oral Surgery*.

[12] Gil J. N., Campos F. E. B., Claus J. D. P., Gil L. F., Marin C., de Freitas S. F. T. (2011). Medial canthal region as an external reference point in orthognathic surgery. *Journal of Oral and Maxillofacial Surgery*.

[13] Altug-Atac A. T., Bolatoglu H., Memikoglu U. T. (2008). Facial soft tissue profile following bimaxillary orthognathic surgery. *Angle Orthodontist*.

[14] Proffit W. R., Proffit W. R., White R. P. (1991). Treatment planning: the search for wisdom. *Surgical Orthodontic Treatment*.

[15] Ahmad Akhoundi M. S., Shirani G., Arshad M., Heidar H., Sodagar A. (2012). Comparison of an imaging software and manual prediction of soft tissue changes after orthognathic surgery. *Journal of Dentistry*.

[16] Epker B. N., Fish L. C. (1986). *Dentofacial Deformities*.

[17] Choi J.-Y., Lee S.-H., Baek S.-H. (2012). Effects of facial hard tissue surgery on facial aesthetics: changes in facial content and frames. *Journal of Craniofacial Surgery*.

[18] Naini F. B., Donaldson A. N. A., McDonald F., Cobourne M. T. (2012). Assessing the influence of chin prominence on perceived attractiveness in the orthognathic patient, clinician and layperson. *International Journal of Oral and Maxillofacial Surgery*.

